# A systematic bibliometric review of clean energy transition: Implications for low-carbon development

**DOI:** 10.1371/journal.pone.0261091

**Published:** 2021-12-03

**Authors:** Wei Zhang, Binshuai Li, Rui Xue, Chengcheng Wang, Wei Cao

**Affiliations:** 1 School of Statistics, Shandong University of Finance and Economics, Jinan, China; 2 Centre for Corporate Sustainability and Environmental Finance, Department of Applied Finance, Macquarie Business School, Macquarie University, Sydney, Australia; 3 School of Humanities and Foreign Languages, Qingdao University of Technology, Qingdao, China; Shenzhen University, CHINA

## Abstract

More voices are calling for a quicker transition towards clean energy. The exploration and exploitation of clean energy such as wind energy and solar energy are effective means to optimise energy structure and improve energy efficiency. To provide in-depth understanding of clean energy transition, this paper utilises a combination of multiple bibliometric mapping techniques, including HistCite, CiteSpace and R Bibliometrix, to conduct a systematic review on 2,191 clean energy related articles obtained from Web of Science (WoS). We identify five current main research streams in the clean energy field, including Energy Transition, Clean Energy and Carbon Emission Policy, Impact of Oil Price on Alternative Energy Stocks, Clean Energy and Economics, and Venture Capital Investments in Clean Energy. Clearly, the effectiveness of policy-driven and market-driven energy transition is an important ongoing debate. Emerging research topics are also discussed and classified into six areas: Clean Energy Conversion Technology and Biomass Energy Utilisation, Optimisation of Energy Generation Technology, Policy-Making in Clean Energy Transition, Impact of Clean Energy Use and Economic Development on Carbon Emissions, Household Use of Clean Energy, and Clean Energy Stock Markets. Accordingly, more and more research attention has been paid to how to improve energy efficiency through advanced clean energy technology, and how to make targeted policies for clean energy transition and energy market development. This article moves beyond the traditional literature review methods and delineates a systematic research agenda for clean energy research, providing research directions for achieving low-carbon development through the clean energy transition.

## 1 Introduction

Currently, many countries worldwide have proposed and implemented their green recovery plans [1–3]. Public voices for transitioning to clean energy are increasingly high, shifting investors’ attention from traditional energy markets to clean energy markets. Therefore, it is important and urgent to systematically understand how to transition to a “clean” world.

Considering this context, the key research question of this study is to provide a comprehensive understanding of the current progress of the clean energy transition and illustrate a research agenda for emerging areas that await more academic and practical attention. To address the research question, this study provides a systematic literature review of 2,191 articles on clean energy related areas obtained from the Web of Science (WoS) Core Collection database over the period from 1950 to 2020. Using a combination of multiple bibliometric mapping techniques, we identify the main streams of current research and propose important topics for future research, providing comprehensive insights for the developments in clean energy transitions and a theoretical basis for more effective ways to achieve carbon neutrality.

Current main streams of clean energy research identified by bibliometric analysis include Energy Transition, Clean Energy and Carbon Emission Policy, Impact of Oil Price on Alternative Energy Stocks, Clean Energy and Economics, and Venture Capital Investments in Clean Energy.

Specifically, the Energy Transition research stream focuses on the barriers to energy transition at the national and household level [4]. Given the governments’ dominant role in promoting the clean energy transition [5], the Clean Energy and Carbon Emission Policy stream concentrates on assessing governments’ related policies and their impacts on carbon emissions. The Impact of Oil Price on Alternative Energy Stocks stream centres around the influencing factors on clean energy stock prices; existing studies show that oil prices, technology stock prices, and interest rates are prominent factors affecting clean energy stock prices [6]. The Clean Energy and Economics stream tends to apply econometric models to test the causal relationship between clean energy consumption and socio-economic variables such as economic growth [7] and foreign direct investment (FDI) [8]. As the soaring demand for clean energy attracts a significant amount of venture capital inflows, especially the private ones [9], the identification and minimisation of investment risk for investors remains the major topic for current research in Venture Capital Investments in Clean Energy.

We further employ the cluster analysis of articles published in recent five years (2015–2020) to propose the emerging trends and future directions in clean energy research. Clean Energy Conversion Technology and Biomass Energy Utilisation, Optimisation of Energy Generation Technology, Policy-Making in Clean Energy Transition, Impact of Clean Energy Use and Economic Development on Carbon Emissions, Household Use of Clean Energy, and Clean Energy Stock Markets are trending topics in the clean energy transition.

Specifically, a growing trend in Clean Energy Conversion Technology and Biomass Energy Utilisation aims to enhance the efficiency and reliability of the biomass gasification system [10, 11]. Research in Optimisation of Energy Generation Technology has been paying more attention to explore ways to effectively integrate new energy resources with traditional ones, construct an efficient hybrid energy system, and resolve the environmental problems incurred from the use of clean energy [12, 13]. Because of the significant discrepancies in the influences of local governments’ clean energy policies [14, 15], the Policy-Making in Clean Energy Transition research continues to explore how local governments should formulate policies conducive to the development of clean energy. The Impact of Clean Energy Use and Economic Development on Carbon Emissions stream provides policymakers with emission reduction recommendations. It starts to investigate the implications of clean energy use and various economic factors, particularly on carbon productivity and carbon transfer [16]. The vital issue of Household Use of Clean Energy research is to increase the the heating system’s energy efficiency and to accelerate the energy transfer of clean cooking [17]. Finally, studies on Clean Energy Stock Markets examine the correlation between clean energy stock prices and the overall stock market, green bond market, electricity market, and coal market [18, 19].

Through systematic reviews of current and trending topics in clean energy research, we aim to delineate a critical research agenda for clean energy transition as an effective way to achieve a low-carbon development and carbon neutrality. The article proceeds as follows. Section 2 introduces the literature retrieval process, the bibliometric techniques used and the descriptive information of existing literature on clean energy. Section 3 illustrates the citation map to identify current main streams in clean energy research and provides a critical review of every stream. Section 4 proposes emerging areas and trending topics. Section 5 concludes the article and provides an agenda for future research in the clean energy transition.

## 2 Research methods

### 2.1 Literature retrieval process

**Fig 1:**
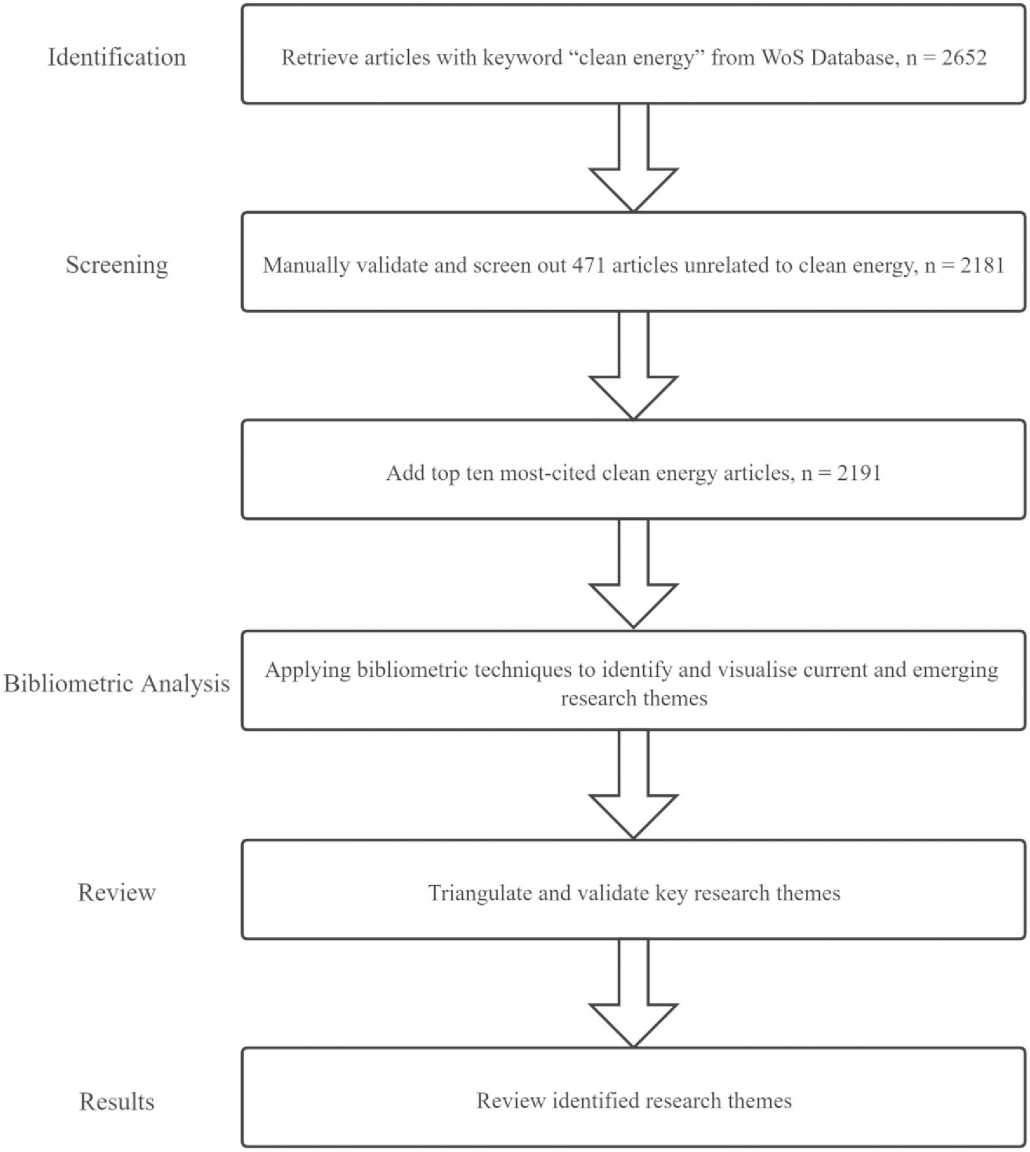
Flow chart of main method steps.

The method of literature retrieval and bibliometric analysis used in this study is illustrated in Fig 1. Specifically, we collect basic information and cited references of clean energy articles from Web of Science (WoS) over the period of 1950 to June 2020, with themes limited to “clean energy” and journal sources limited to “SSCI, SCIE, A&HCI.” A total of 2,652 initial articles is retrieved. For validation purposes, we have implemented manual checks to select relevant articles, resulting in 471 irrelevant articles removed. Following Linnenluecke et al. (2017) [20], we then add another ten most cited clean energy articles into our database. Therefore, we obtain 2,191 articles in our final dataset.

Table 1 shows the basic information of sample articles. The next section will introduce the bibliometric techniques used, i.e., R Bibliometrix, HistCite and CiteSpace, to analyse these clean energy articles.

**Table 1:** Basic information.

Description	Observations
Source journals	298
Documents	2,191
Average citations per document	24
References	81,825
Keywords	6,682
Authors	6,496

### 2.2 Bibliometric techniques

#### 2.2.1 R Bibliometrix

Bibliometrix is a widely-used R-package developed by Massimo and Corrado (2017) [21]. It provides access to a wide range of bibliometric functions and excellent visualisation tools. This article uses Bibliometrix to carry out descriptive statistical analysis to illustrate the diagrams for the number of publications over time and the author-keyword-journal connections (Sankey diagram).

#### 2.2.2 HistCite

HistCite is a citation software developed by Eugene (2004) [22]. The citation map generated by HistCite is highly useful for mapping out the relationships among highly cited publications [23]. It is a popular tool for researchers to explore research hotspots and how research themes develop over time. It is an essential tool for bibliometric analysis. This paper utilises HistCite to generate the citation map of 50 highly cited articles as guidance to identify key streams of clean energy research.

#### 2.2.3 CiteSpace

CiteSpace is a Java visualization application developed by Chen (2017) [24]. It has powerful bibliometric and visualization functions and is extremely popular in research. It generates a spectrum of colors to depict the literature network’s temporal orders and uses algorithms such as LLR for cluster labeling extraction. This article uses this application to cluster keywords of relevant literature from 2015 to 2020 to identify future research hotspots.

### 2.3 Descriptive information

#### 2.3.1 Publications over time

**Fig 2:**
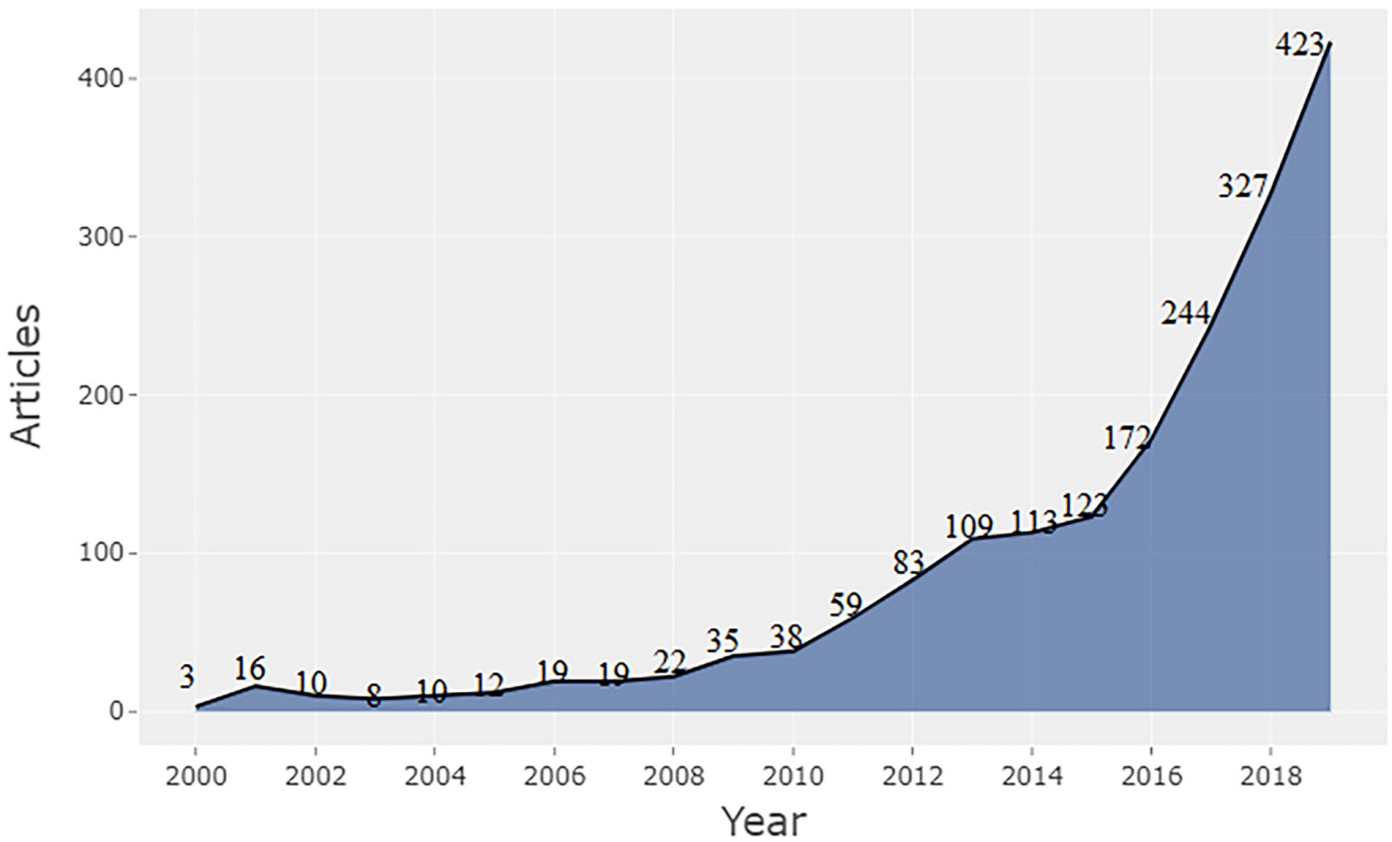
Number of publications over 2000–2019.

Fig 2 illustrates the number of publications from 2000 to 2019. The sample ends at June 2020 and the total number of publications from January 2020 to June 2020 is 274; so to make the diagram more illustrative, we do not include the publication number of 2020. Fig 2 indicates a three-stage development of clean energy research. The first stage (from 2000 to 2010) is the initial stage, with an average of 17.5 articles published per year. The period of 2011–2015 is the developing stage, with an average of 97.4 articles published per year. The publications in the clean energy areas experience a significant increase from 2016, with an average number of 291.5 publications per year (2016–2019). It signals a robust momentum in clean energy research. The clean energy transition is crucially important to mitigate climate change issues and achieve carbon neutrality. Therefore, it is expected to continue to (exponentially) grow in the next few decades.

#### 2.3.2 Author-Keyword-Journal (AKJ) analysis

**Fig 3:**
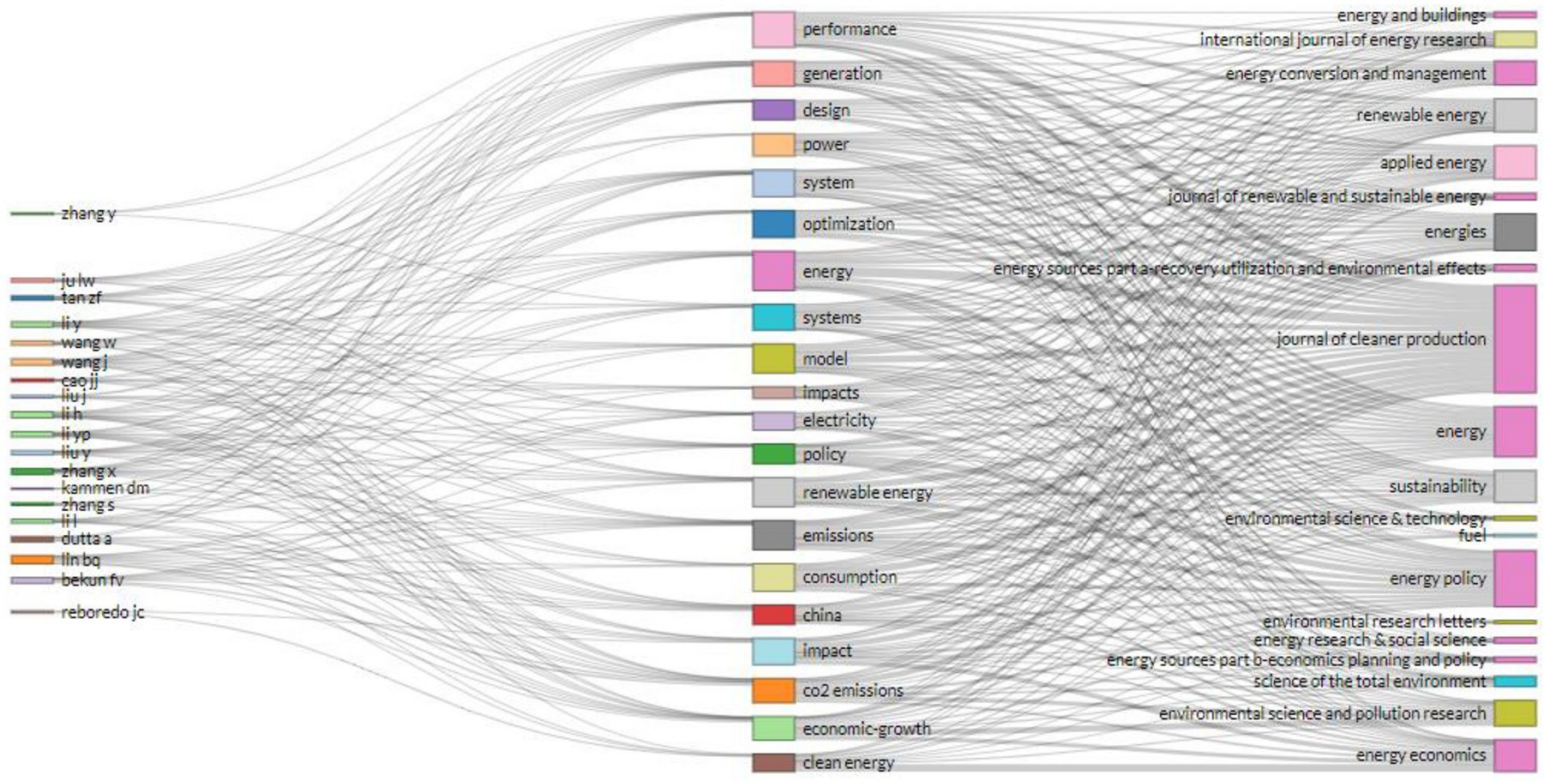
Sankey diagram.

Fig 3 displays the Sankey diagram, i.e., the author-keyword-journal diagram. The three columns in Fig 3 are the top 20 authors, keywords, and source journals in clean energy research, respectively. The Sankey diagram gives a graphical overview of influential clean energy research. The keywords broadly fall into the following categories: Clean Energy Stock Performance, Clean Energy and Economy Growth, Energy Consumption and Carbon Emissions, Clean Energy Power Generation, and Clean Energy Policy. The major publishing journals in the clean energy area include *Renewable Energy*, *Journal of Cleaner Production*, *Energy Policy*, *Energy Economics*, *Applied Energy*, etc.

## 3 Developments in clean energy transition research

### 3.1 Identification of current research streams

**Fig 4:**
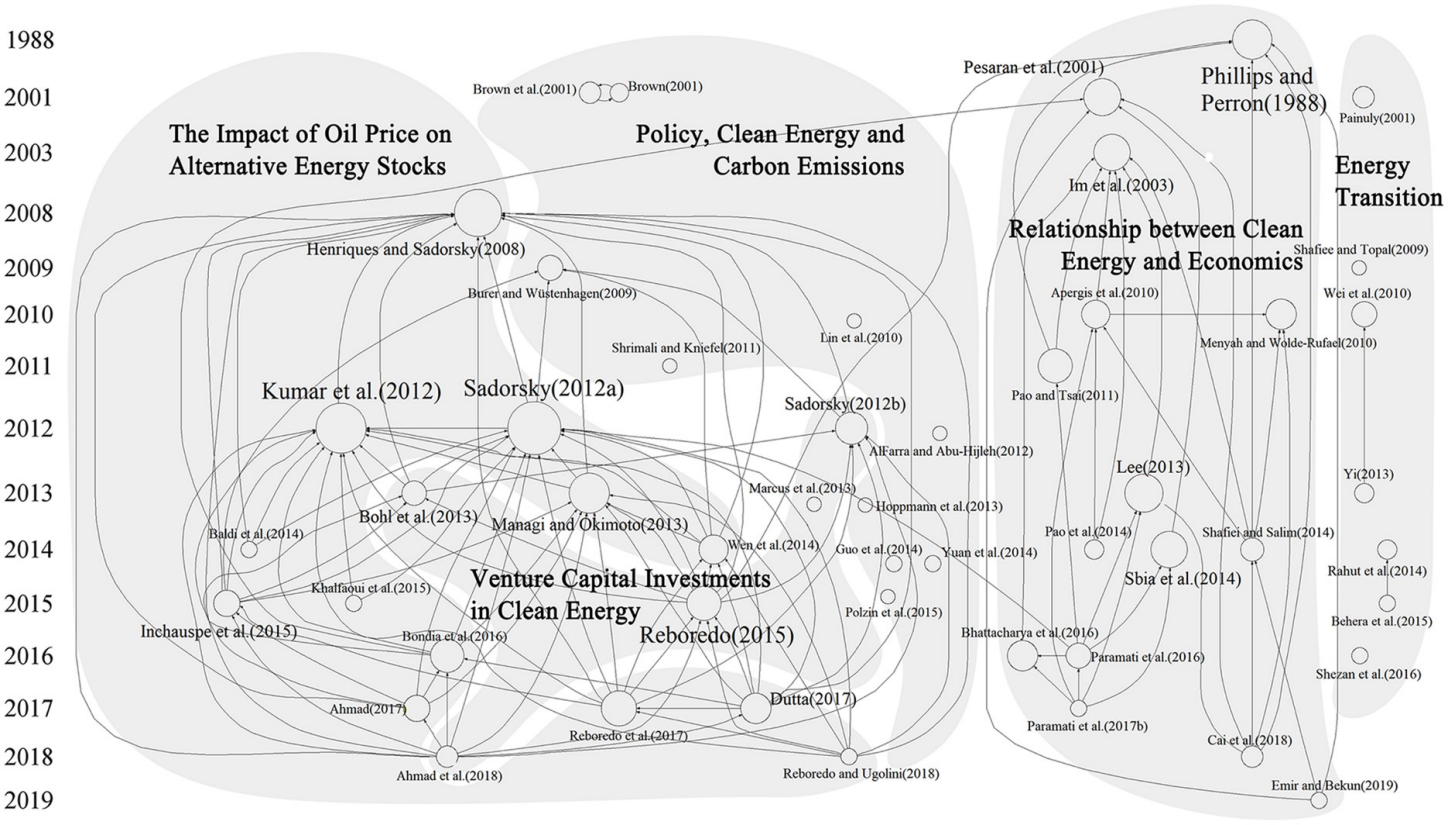
Citation network map of highly cited articles.

In this section, we utilise HistCite to generate a citation network map for the top 50 cited articles in clean energy transition research. We then apply the triangulation process [23] to assign titles for each research stream, laying the foundation for the systematic review of these research themes. Table 2 summarises the citation information of top-cited literature, and Fig 4 illustrates the corresponding citation network map.

**Table 2:** Top 50 cited articles.

#	Author	Journal	LCS	GCS
1	Phillips and Perron (1988)	Biometrika	34	5,856
2	Pesaran et al. (2001)	Journal of Applied Econometrics	31	3,814
3	Painuly (2001)	Renewable Energy	10	385
4	Brown (2001)	Energy Policy	8	64
5	Brown et al. (2001)	Energy Policy	10	247
6	Im et al. (2003)	Journal of Econometrics	29	4,375
7	Henriques and Sadorsky (2008)	Energy Economics	49	203
8	Shafiee and Topal (2009)	Energy Policy	5	726
9	Bürer and Wüstenhagen (2009)	Energy Policy	14	201
10	Wei et al. (2010)	Energy Policy	14	267
11	Menyah and Wolde-Rufael (2010)	Energy Policy	21	308
12	Lin et al. (2010)	Energy Policy	5	81
13	Apergis et al. (2010)	Ecological Economics	18	272
14	Pao and Tsai (2011)	Energy	26	328
15	Shrimali and Kniefel (2011)	Energy Policy	5	83
16	Kumar et al. (2012)	Energy Economics	56	116
17	Sadorsky (2012a)	Energy Economics	61	190
18	Sadorsky (2012b)	Energy Policy	23	48
19	AlFarra and Abu-Hijleh (2012)	Energy Policy	5	36
20	Marcus et al. (2013)	Organization & Environment	5	29
21	Lee (2013)	Energy Policy	32	156
22	Bohl et al. (2013)	Energy Economics	13	38
23	Yi (2013)	Energy Policy	9	57
24	Hoppmann et al. (2013)	Research Policy	5	106
25	Managi and Okimoto (2013)	Japan and the World Economy	35	68
26	Sbia et al. (2014)	Economic Modelling	29	165
27	Wen et al. (2014)	Energy Economics	19	40
28	Yuan et al. (2014)	Energy Policy	6	62
29	Baldi et al. (2014)	Energy Policy	6	30
30	Shafiei and Salim (2014)	Energy Policy	12	224
31	Pao et al. (2014)	Energy Policy	9	42
32	Rahut et al. (2014)	Energy	9	44
33	Guo et al. (2014)	Energy Economics	6	73
34	Reboredo (2015)	Energy Economics	26	61
35	Inchauspe et al. (2015)	Energy Economics	15	32
36	Khalfaoui et al. (2015)	Energy Economics	6	84
37	Polzin et al. (2015)	Energy Policy	5	107
38	Behera et al. (2015)	Energy	6	31
39	Bhattacharya et al. (2016)	Applied Energy	20	293
40	Bondia et al. (2016)	Energy	24	51
41	Paramati et al. (2016)	Energy Economics	14	81
42	Shezan et al. (2016)	Journal of Cleaner Production	6	73
43	Paramati et al. (2017b)	Energy Economics	6	42
44	Reboredo et al. (2017)	Energy Economics	27	63
45	Dutta (2017)	Journal of Cleaner Production	21	37
46	Ahmad (2017)	Research in International Business and Finance	15	21
47	Cai et al. (2018)	Journal of Cleaner Production	10	61
48	Ahmad et al. (2018)	Economic Modelling	10	14
49	Reboredo and Ugolini (2018)	Energy Economics	6	11
50	Emir and Bekun (2019)	Energy & Environment	6	48

Note: LCS is abbreviated for local citation score, representing the number of citations by other 2,190 sample articles; GCS is abbreviated for global citation score, representing the number of citations by all other articles from WoS.

In Fig 4, each article is displayed as a node, with a larger-sized node denoting a higher number of citations. The arrows illustrate the citation connections among articles, with arrowheads pointed to the cited ones. Through the triangulation process, we categorise the current clean energy research into the following streams: Energy Transition, Clean Energy and Carbon Emission Policy, Impact of Oil Price on Alternative Energy Stocks, Clean Energy and Economics, and Venture Capital Investments in Clean Energy. In the next section, we provide a comprehensive review of each of these five research streams.

### 3.2 Review of main research streams

#### 3.2.1 Energy transition

The transition from traditional energy towards clean energy remains the major challenge for the first half of the 21st century [4]. We discuss the Energy Transition stream from two perspectives: obstacles in clean energy transition and influencing factors on household energy transition.

*3*.*2*.*1*.*1 Obstacles in the clean energy transition*. Current major challenges to clean energy transition include subsidies to traditional energy, high initial capital cost, high transaction cost, high financing risk, lack of price risk assessment, lack of clean technology, low market acceptance rate, and immature regulatory systems [25–28]. Luthra et al. (2015) [29] categorised 28 obstacles to the clean energy transition into seven dimensions: economy and finance, market, awareness and information, technology, ecology and geography, culture and behavior, political and government issues. For an in-depth look, the more challenging obstacles are ecological problems, consumers’ lack of awareness of clean technology, inability to obtain solar radiation data, technical complexity, rehabilitation disputes and lack of political commitment.

*3*.*2*.*1*.*2 Influencing factors on household energy transition*. Household energy use is a substantial part of energy consumption. Investigating the driving factors affecting household energy transition is an effective way to promote clean energy transition. Researchers conduct surveys on households in urban and rural areas in China, India, Brazil, Ethiopia, Guatemala, and other countries. Their results show that 1) household income and fuel prices are the dominant factors affecting household energy transition, 2) household size, household members’ occupations, and education levels are also important factors, and 3) the availability and cost of clean energy alternatives have a significant impact on rural household energy transition [30–42].

#### 3.2.2 Clean energy and carbon emission policy

The high carbon energy represented by raw coal was still the main factor in promoting the growth of energy-related CO2 emissions [43]. Appropriate and effective policies are needed to accelerate the clean energy transition. The majority of countries worldwide have set goals to increase the share of clean energy consumption and reduce greenhouse gas (GHG) emissions, resulting in various supportive policies [44]. Existing policies concentrate around quantity-driven policies. For instance, levying a carbon tax is a typical quantity-driven policy. Guo et al. (2014) [5] argues that a moderate carbon tax significantly reduces carbon emissions and fossil fuel consumption, with a minimal impact on economic growth. But a more recent study claims that carbon taxes are not always good for the environment [45]. Another example is feed-in tariffs (FIT), a quantity-driven policy targeted at specific technology [46]. It is generally regarded as an effective policy for clean energy transition due to its advantages of low costs, low risks, and high innovation incentives [47–51].

#### 3.2.3 Impact of oil price on alternative energy stocks

The way how oil prices affect stock prices works as follows. On the one hand, rising oil prices increase production and service costs and decrease cash flow turnover, leading to a stock price drop. On the other hand, rising oil prices also indicate the mounting inflation pressure and discount rate, resulting in stock price drop [52]. As a critical component of the stock market, energy stocks are also highly correlated with oil prices [52–55]. Nevertheless, the negative impact of oil prices may only be a short-term effect for clean energy stocks [6].

#### 3.2.4 Clean energy and economics

The clean energy transition is closely related to economic development [7]. In Fig 4, the theme of Clean Energy and Economy contains comparatively more nodes (articles), the majority of which use different econometric models to examine the relationship between clean energy consumption and socio-economic variables such as economic growth and FDI. In the short term, there exists a positive correlation and bidirectional causal relationship between clean energy consumption and economic development. In the long run, clean energy consumption will positively affect on economic growth [8, 56–60]. The empirical results of Paramati et al.(2016) [8] indicate that there is a unidirectional causality running from FDI to clean energy consumption, with inflows of FDI having a positive impact on the latter. Moreover, the results of Paramati et al.(2016) [8] also show that the development of the stock market has brought more investment in the clean energy industry and plays a significant role in promoting clean energy transition.

#### 3.2.5 Venture capital investments in clean energy

Venture capital (VC) is one of the main drivers of technology advancement, especially in new and innovative fields such as clean energy. As the demand for clean energy increases, there has been a surge of venture capital inflows, especially private VCs, into clean energy companies [9, 61, 62]. Currently, clean energy has become the third-largest venture investment field [63]. In addition, there are also risks embedded in clean energy investments, including market risks, technology risks, human resource risks, and more importantly, regulatory risks [64]. However, it is feasible to reduce market risks through appropriate business models, reduce technology risks through publicly funded R&D projects, reduce human resource risks through market liberalisation, and reduce regulatory risks through effective government policies [64, 65].

## 4 Emerging research areas

To illustrate the emerging topics in clean energy transition research, we utilise CiteSpace to conduct cluster analysis on sample articles published in recent five years, from 2015 to 2020. The following two sections provide basic information on identified emerging topics and provide a detailed analysis of the relevant literature.

### 4.1 Identifications of emerging research areas

**Fig 5:**
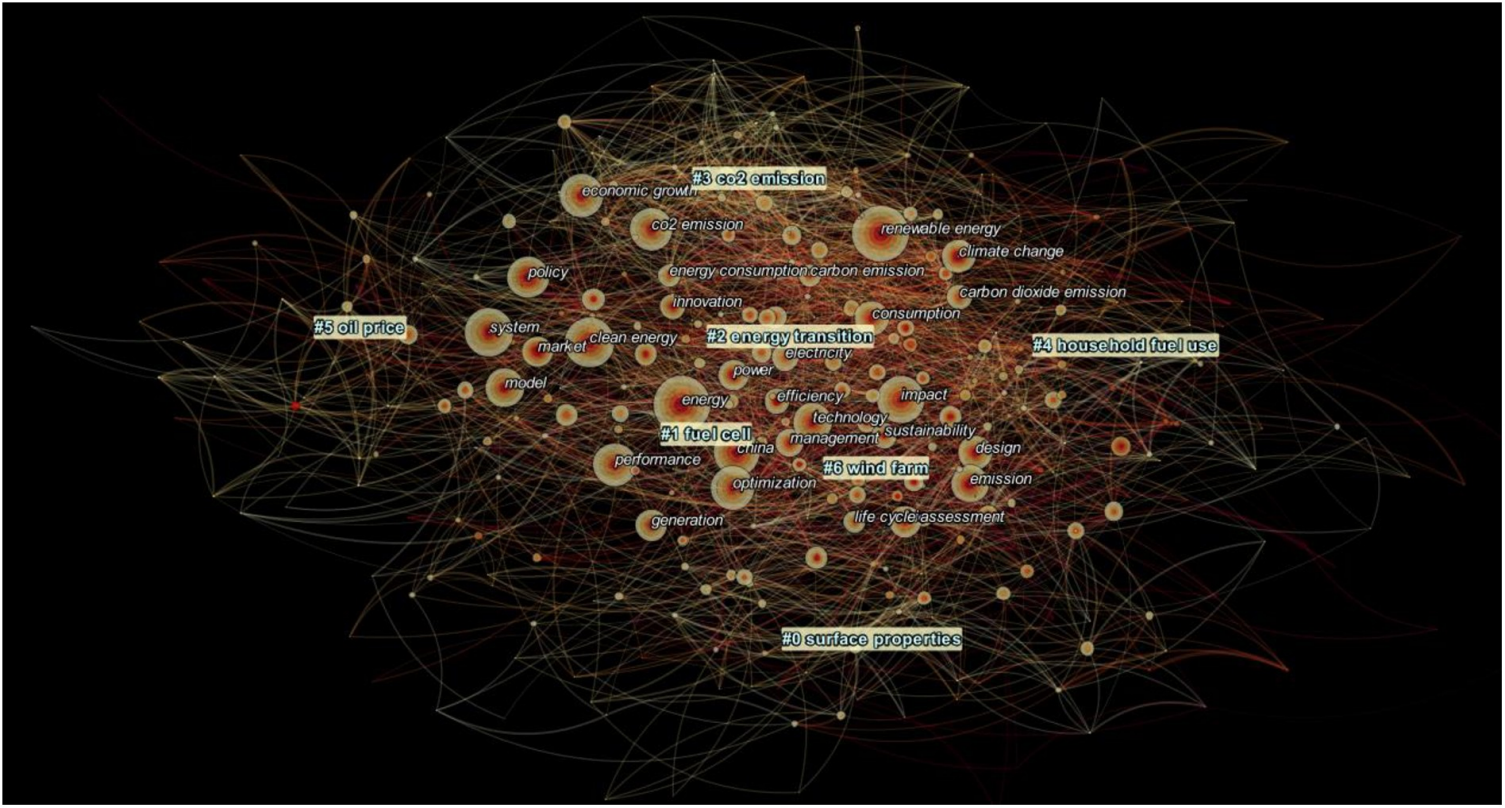
Keyword co-occurrence network map of 2015–2020 publications.

Fig 5 demonstrates the keyword co-occurrence network map of recent five years’ publications in clean energy transition areas, with a larger circle (keyword) representing more frequent occurrence, and darker colour representing earlier occurrence (publication time). The lines connecting circles (keywords) refer to co-occurrence.

Using cluster analysis, CiteSpace classifies recent five years’ publications into seven clusters, reflecting seven emerging research topics in clean energy research. The clustered emerging topics include Surface Properties, Fuel Cell, Energy Transition, CO_2_ Emission, Household Fuel Use, Oil Price, and Wind Farm. Once again, we apply the triangulation process to define the title of each cluster (area) and provide more details in Table 3.

**Table 3:** Summary of emerging research areas clusters 1 and 6 have large common features and are thus combined together as “Optimisation of Energy Generation Technology” by triangulation process.

Cluster ID	Cluster Title	Size	Silhouette	Year	Keyword-Frequency	Research Area
0	Surface Properties	81	0.691	2017	bioma-68; natural gas-40; biogas-31; CO_2_-31; water-27	Clean Energy Conversion Technology and Biomass Energy Utilisation
1	Fuel Cell	71	0.654	2016	system-172; optimisation-113; design-77; generation-77; management-61	Optimisation of Energy Generation Technology
2	Energy Transition	60	0.709	2016	renewable energy-230; policy-106; technology-89; power-72; electricity-64	Policy Making in Clean Energy Transition
3	CO_2_ Emission	57	0.840	2017	CO_2_ emission-125; economic growth-109; energy consumption-64; carbon emission-56; carbon dioxide emission-52	Impact of Clean Energy Use and Economic Development on Carbon Emissions
4	Household Fuel Use	50	0.738	2017	combustion-34; fuel-34; air pollution-32; cost-28; coal-25	Household Use of Clean Energy
5	Oil Price	38	0.861	2017	clean energy-185; performance-137; model-112; market-60; oil price-35	Clean Energy Stock Markets
6	Wind Farm	35	0.781	2016	energy-177; impact-149; China-126; emission-101; consumption-79	Optimisation of Energy Generation Technology

Note: Size denotes the number of articles included in each cluster; Silhouette denotes within-cluster correlation, ranging from 0 to 1, with a larger value representing a higher correlation; Keyword-Frequency denotes the occurrence frequency of each keyword; and Research Area is the theme for each cluster generated by triangulation process.

### 4.2 Analyses of emerging research areas

#### 4.2.1 Clean energy conversion technology and biomass energy utilization

Converting industrial waste and household garbage into clean energy can help deal with the current shortage of clean energy and protect the environment through the recycling process. Studies show that kitchen waste, animal waste, agricultural waste, forestry waste, waste plastics and waste tyres can be converted into clean energy through advanced technologies such as thermochemical conversion or hydrothermal carbonisation [10, 66–70]. Research on improving these conversion technologies is a trending research hotspot. For example, biomass gasification is a feasible and practical clean energy conversion technology, but it faces crucial challenges to effectively eliminate the tar generated during the gasification process [11, 71, 72]. Another trending research topic in this area is to enhance the efficiency and reliability of biomass gasification. In addition, with the continuous advancement of clean energy conversion technology, how to formulate policies to implement more effective classifications of waste and refuse continues to be an urgent issue to be further explored.

#### 4.2.2 Optimisation of Energy Generation Technology

Comprehensive utilisation of various energy resources is an ideal approach to alleviate the energy crisis [73]. Many scholars have investigated how to integrate various new and traditional energy resources, including photovoltaics, batteries, diesel, wind energy, and solar energy, to build a highly effective hybrid energy system [12, 13, 74]. Research on the development of clean energy battery systems, the optimisation of power station scale, and generator systems also receives extensive academic attention [75, 76].

Electricity generation from clean energy, such as wind and solar, plays a key role in the clean technology optimisation research [77, 78]; however, a series of problems are setting obstacles for it. For instance, wind power generation has a high level of uncertainty, and there are potential exposure risks to the operation of a power grid [79, 80]. Therefore, research on wind power generation in recent years tends to focus on wind flow models with the expectation to achieve a more accurate prediction of wind power generation [79, 81]. Besides, considering the negative impact of the wind power plant on the environment, researchers have made significant explorations on the environmental effects of wind farms and on the selection of wind farm locations for harnessing wind energy [82–87]. Resolving the problems arising from the use of clean energy is an important topic to be further examined.

#### 4.2.3 Policy-making in clean energy transition

Regulations and legislations guarantee the secure transition towards clean energy. The government thus plays an essential role in addressing the potential risks incurred by the clean energy transition process. Relevant policies involve electricity price standards, emission trading system, clean energy investment policies, and the use of innovative finance tools in clean energy support [14, 15, 88, 89]. Tingey and Webb (2020) [90], Bayulgen (2020) [91] and Proedrou (2019) [92] evaluate the practices of local government in the UK, US, and EU in terms of the clean energy transition. Their results indicate that although most local governments have adopted clean energy policies, the effectiveness of these policies varies substantially. To improve the effectiveness of energy policies, the views of different local energy users should be taken into account [93]. Therefore, what policies local governments should formulate to accelerate clean energy development will continue to be one of the research hotspots in clean energy transition research.

#### 4.2.4 Impact of clean energy use and economic development on carbon emissions

A large body of literature concentrates on how clean energy, economic growth, land resource use, industrial restructuring, financial market development, the application of new technology and R&D activities affect carbon emissions in recent clean energy areas [16, 94–100]. And it is likely to be a hot issue worth studying in the future. With the improvement of carbon emission measurement methods, research on the impact of the aforementioned factors on carbon productivity and carbon transfer is attracting increasing scholarly attention [101–103]. Moreover, from a micro point of view, the role of enterprises, as an essential component of the national economy, in environmental governance will become another trending research direction [104].

#### 4.2.5 Household use of clean energy

Given that household energy use for heating and cooking is an essential part of energy use, recent studies have made substantial progress on enhancing the heating system’s energy efficiency and advancing the clean energy transition for cooking [17, 105–108]. Moreover, in terms of the driving factors on the household clean energy transition, more recent literature indicates that household income and energy prices are found to have significant effects on household energy use decisions. Therefore, energy poverty is also an issue worth future research attention [109–111].

#### 4.2.6 Clean energy stock markets

Without support from the financial markets, the clean energy industry alone cannot secure the desired level of clean energy development. In effect, clean energy stocks have recently become a popular investment asset for investors, especially for those with strong considerations for environmental protection [18, 112]. In addition to the follow-up research on the impact of oil price on clean energy stock prices [19, 113, 114], increasingly great attention has been focused on the relationship between clean energy stock investment and its driving factors, including the overall stock market, bond market, electricity market, coal market, gold market, silver market and many more [18, 112, 115–118]. Therefore, we reckon that the relationship between clean energy stocks and the financial markets, especially the green bond market [119] and the carbon market [53], has great potential to be explored in future clean energy research.

## 5 Conclusions

Clean energy transition plays a crucial role in post-pandemic green recoveries and carbon neutrality. To advance understanding of clean energy transition, this paper provides a systematic review of existing clean energy literature through a combination of bibliometric analysis techniques. Overall, there has been a surging trend of clean energy research since 2000, especially after 2016, clean energy research has experienced exponential growth.

We collect clean energy literature from the Web of Science (WoS) Core Collection database over the period from 1950 to 2020. Using bibliometric analysis, we identify and provide a comprehensive review of five current main research streams in the clean energy area, including Energy Transition, Clean Energy and Carbon Emission Policy, Impact of Oil Price on Alternative Energy Stocks, Clean Energy and Economics, and Venture Capital Investments in Clean Energy. Main challenges and opportunities facing the current clean energy transition with respect to each research stream are investigated.

To illustrate emerging research topics that attract more recent academic attention, we apply bibliometric cluster analysis to clean energy literature published in recent five years (from 2015 to 2020). Six trending research areas in the clean energy field are proposed and analysed, including Clean Energy Conversion Technology and Biomass Energy Utilisation, Optimisation of Energy Generation Technology, Policy-Making in Clean Energy Transition, Impact of Clean Energy Use and Economic Development on Carbon Emissions, Household Use of Clean Energy, and Clean Energy Stock Markets.

Future research agenda of clean energy awaits theoretical and practical exploration. We propose that the advancement of clean technology is at the heart of clean energy transition and post-pandemic green recovery. Funding for clean energy transition is a critical challenge that needs innovative financial instruments and policy support. Thus green bond markets, carbon taxes and emission trading system (ETS) need in-depth investigation. With more disruptive financing tools available such as crowdfunding, efforts from enterprises and individuals also deserve more attention. In addition, international collaborations on clean energy transition projects are highly recommended. Intensive international collaborations and cooperations are of high importance to achieve the low-carbon development. The completion of the global warming goal needs collective contributions from all countries over the world. A community of common destiny for all of humankind cannot be successfully built with efforts from only a small number of highly engaged countries. The current collaboration in clean energy research lacks worldwide collaborations in climate change actions. Therefore, it is highly recommended that all countries shall shoulder their responsibilities in climate change mitigation and adaptation, with steady growth of environmental investments and frequent collaborations with leading countries in climate change actions.
